# Weaning Time in Preterm Infants: An Audit of Italian Primary Care Paediatricians

**DOI:** 10.3390/nu10050616

**Published:** 2018-05-15

**Authors:** Maria Elisabetta Baldassarre, Antonio Di Mauro, Annarita Pedico, Valentina Rizzo, Manuela Capozza, Fabio Meneghin, Gianluca Lista, Nicola Laforgia, on behalf of Italian Society of Pediatrics (SIP), Italian Society of Neonatology (SIN), Italian Society of Pediatric Gastroenterology, Hepatology, and Nutrition (SIGENP) and Italian Federation of Paediatricians (FIMP)

**Affiliations:** 1 Neonatology and Neonatal Intensive Care Unit, Department of Biomedical Science and Human Oncology, “Aldo Moro” University of Bari, 70124 Bari, Italy; antonio.dimauro@uniba.it (A.D.M.); annaritapedico92@gmail.com (A.P.); rizzo.vale@libero.it (V.R.); manuelacapozza26@gmail.com (M.C.); nicola.laforgia@uniba.it (N.L.); 2 Member of SIGENP (Italian Society of Pediatric Gastroenterology, Hepatology, and Nutrition), via Libero Temolo 4 (Torre UB), 20126 Milan, Italy; 3 Neonatology and Neonatal Intensive Care Unit, Department of Paediatrics, Ospedale dei Bambini “V.Buzzi”, ASST FBF SACCO, 20154 Milano, Italy; meneghin.fabio@hsacco.it (F.M.); gianluca.lista@asst-fbf-sacco.it (G.L.)

**Keywords:** preterm newborn, weaning, primary care

## Abstract

Introduction: According to the 2016 Italian National Institute of Statistics (Istat) data in Italy, about 6.7% of all newborns are born prematurely. Due to the lack of data on current complementary feeding in preterm infants in Italy, the aim of the survey was to evaluate individual attitudes of primary care paediatricians, concerning the introduction of complementary foods in preterm infants. Methods: An internet-based survey was conducted among primary care paediatricians, working in Italy, regarding (1) timing of the introduction of complementary foods to preterm newborns; (2) type of complementary foods introduced; (3) vitamin D and iron supplementations. Results: A total of 347 primary care Italian paediatricians answered the questionnaire; 44% of responders based the timing of the introduction of solid food exclusively on an infant’s age, 18% on an infant’s neurodevelopmental status and 4% on the body weight; the remaining 34% based the timing on two or more of these aspects. The type of complementary foods did not comply with an evidence-based sequence; 98% of participants promoted vitamin D supplementation and 89% promoted iron supplementation with great diversity in timing and doses. Conclusions: Due to limited evidence, there is a great heterogeneity in the attitudes of primary care paediatricians concerning the introduction of complementary foods to preterm newborns. Further research is needed to provide evidence-based guidelines regarding weaning preterm newborns.

## 1. Introduction

The term “complementary food” refers to solids and liquids, other than breast milk and infant formula, needed when milk is no longer adequate to meet all infant’s nutritional requirements [1]. Despite there being widely endorsed recommendations and increasing evidence for full-term newborns, that postnatal growth rate has significant long-term consequences [2,3] and might be influenced by timing and type of solid foods which are introduced [4,5,6], there, surprisingly, is no evidence-based guidelines in preterm infants. Only few position papers are available, with controversial recommendations [7,8,9,10,11]. Recently, the European Society of Pediatric Gastorenterology Hepatology and Nutrition (ESPGHAN) published a position paper on complementary feeding providing no recommendations regarding preterm weaning to health care professionals [12].

This is a matter of serious concern because there is clear evidence that preterm infants are at a higher risk of postnatal growth deviation, including rapid catch-up growth or failure to thrive.

The optimal duration of milk feeds alone (exclusive breastfeeding or formula or a combination of the two) has not yet been determined in preterm infants, mainly because of the lack of scientific data. A paucity of evidence suggests that an early start of complementary food might provide higher caloric density and iron reserves that may better meet the increased requirements of preterm infants [13]. On the other hand, a recent study suggested that early introduction of solid food may be related to a higher rate of hospital readmission with no difference in growth compared to delayed introduction of complementary food [14].

For this reason, a review of randomized clinical trials (RCTs) or quasi-randomised trials on the effect of nutrition education for weaning infants born preterm is being assembled by the Cochrane Neonatal Group [15]. In 2002, Norris et al. examined current infant feeding practices among caregivers of 253 preterm born infants, enrolled in Southeast England, over a 2-year period [16]. The study showed that the introduction of complementary foods varied widely, and the absence of scientific evidence has led to different and contrasting approaches by caregivers, both in too early or too late weaning.

To the best of our knowledge, no data on current complementary feeding in preterm infants is available in Italy.

## 2. Aim

According to the 2016 Italian National Institute of Statistics (Istat) data, around 6.7% of babies are born preterm in Italy, and approximately 0.9% are born extremely preterm (<28 + 0 weeks’ gestation). In 2015, this amounted to about 32,000 babies born preterm and 4300 born extremely preterm. Due to the lack of data on current complementary feeding in preterm infants in Italy, the aim of the study was to investigate the attitude of Italian primary care paediatricians regarding the introduction of complementary foods and other supplementations to preterm infants.

## 3. Methods

From May 2016 to May 2017 a survey was conducted in Italy among primary care paediatricians.

An internet-based questionnaire was designed using Google Forms [17].

In total, 1000 primary care paediatricians were randomly selected from the mailing list of the Italian Federation of Paediatricians (Federazione Italiana Medici Pediatri, FIMP) and invited to participate anonymously in the study.

The participation was voluntary and written consent was not needed. Ethical approval was not requested because all data were anonymous.

The questionnaire, consisting of 34 items, included 4 areas of interest: (1) demographical characteristics (gender, residence and age) of primary care paediatricians; (2) timing of introduction of complementary foods; (3) type of foods introduced and (4) timing and dosage of vitamin D and iron supplementation for infants born less than 34 weeks of gestation.

Factors affecting the introduction of complementary foods (age, infant’s neurodevelopmental status and body weight) were investigated. Both chronological age and corrected age was considered when recording the timing of introduction of complementary foods to preterm infants.

The database was uploaded as an Excel file and data was analysed by Stata MP11 software (StataCorp LLC 4905 Lakeway Drive College Station, TX, USA).

Categorical variables were reported as proportions. Quantitative variables were described as means ± standard deviations (SD) and median.

## 4. Results

A total of 347 primary-care Italian paediatricians (Male: 112; Female: 235; mean age: 56 years old) returned the questionnaire. 

According to our data, the paediatric approach regarding the introduction of solid foods was exclusively based on the infants’ age for 44% of responders, based only on neurodevelopmental status for 18% and based only on body weight for 4% of responders. The remaining 34% decided that the timing of solid food introduction should be based on two or more of these categories collectively.

Regarding the infants’ age for food introduction, 58% of paediatricians suggested weaning at 5.5 (0.58) months of life for corrected age and 42% at 5.6 (0.50) months for chronological age.

Regarding types of food at the start of weaning, 37% of responders recommended a complete meal with cereals, pureed meat, vegetables and fruits; 36% of responders recommended only pureed fruits; 27% of responders recommended only cereals with pureed vegetables. Refer to Table 1 below.

Twenty-seven percent of responders suggested to postponing allergenic foods such as fish and eggs in preterm infants.

Ninety-eight percent of participants gave vitamin D supplementation and 91% of participants gave iron supplementation to preterm infants, but with many differences concerning timings to start and stop (Table 2) as well as dosage (Table 3).

## 5. Discussion

In the last few decades, improvements in neonatal management, both during hospitalization and follow-up, has resulted in a significant increase in survival of very preterm infants. An adequate nutritional status of preterm infants has been considered one of the most important goals for neonatal management during both hospitalization and follow-up after discharge [18].

During the first year of life the paediatrician has a fundamental role to promote the ‘catch-up growth’ of preterm infants in accordance with the standard reference [19]. In this period, it is not only important to suggest the choice of the best “milk” (human milk with or without supplementation and/or formula) to enhance a balanced infant growth but also to choose the right food and right time to start weaning, dependent upon family compliance. To the best of our knowledge, information on current weaning practices in the preterm infant population in Italy is not available. This is the first study aimed to investigate the attitude of the Italian primary care paediatricians with regard to the introduction of complementary foods to preterm born infants.

The Joint Consensus Statement on weaning preterm infants suggests “preterm infants should be considered for weaning between 5 and 8 months uncorrected age to ensure that sensitive periods for the acceptance of solids are not missed and to allow development of appropriate feeding skills” [20].

Our survey showed that less than 50% of paediatricians consider the “chronological—uncorrected—age as the key-point for weaning in preterm born infants and this choice is in agreement with this “consensus”. However, in extremely preterm newborns (<24 weeks of gestational) it is important to evaluate not only chronological age but also neurological development. In our results, more than 50% of the paediatricians started weaning according to the infant’s neurodevelopmental status. This answer is toward complete agreement with the The Joint Consensus Statement on weaning preterm infants that suggests considering the “*degree of motor development*” for the decision to start weaning.

Our data indicated that in some cases a combination of the different criteria for weaning preterm born infants (e.g., uncorrected age, neurological development and body weight) has been used. We think that this choice could be a good answer in reducing “weaning failure” and to increase parents’ compliance.

Our paediatricians did not choose the same type of foods to start weaning, i.e., complete meal or single type of food. This behaviour reflects the absence of clear published data and might not meet the nutritional needs of growing preterm infants. Furthermore, despite in terms of allergy prevention there is no benefit in delaying the introduction of potentially allergenic foods, Italian primary care paediatricians trend to defer the introduction of some type of complementary foods.

Since many preterm infants are at risk of anaemia and osteopenia, we investigated the paediatricians’ approach of vitamin D and iron supplementation.

Even though 98% of participants promoted vitamin D supplementation and 91% of participants promoted iron supplementation in preterm infants, we found a great heterogeneity concerning time (start and end) and doses.

Regarding vitamin D supplementation, in a commentary from ESPGHAN on Enteral Nutrient Supply for Preterm Infants [21], vitamin D intake of 800–1000 IU/day during the first months of life was recommended to support neuromuscular function and bone mineralisation. However, only 10% of our responders followed this recommendation.

In the same document by ESPGHAN Committee on Nutrition, regarding iron supplementation, an intake of 2–3 mg/kg/day is recommended and should be started at 2–6 weeks of age. Our results show that 39% of responders recommended giving an inadequate dose, while 3% suggested a too high dose, which could result in a significant risk of retinopathy of prematurity.

## 6. Conclusions

In the presence of a lack of a general consensus, primary-care paediatricians have very different approaches to the weaning of healthy preterm infants.

This diversity in weaning practice could lead to nutritional compromise that the preterm infant is poorly equipped to withstand.

This study highlights the need for the development of clear and evidence-based practical guidelines on appropriate complementary feeding practices in preterm infants (time, type and sequence of foods) for health care professionals and families of preterm born infants.

We believe that, mainly for Very Low Preterm Newborns (VLBW) and Extremely Low Preterm Newborns (ELBW) infants, who are frequently affected by co-morbidities (i.e., bronchopulmonary dysplasia, cerebral damage) customized instructions shared amongst the primary-care paediatricians and the follow-up service of the birth hospital are needed, mainly based on neurodevelopmental status respect to age or weight.

Our study shows some limitations. Despite internet-based surveys are increasingly used for data collection, this methodology has produced a number of factors that influence data quality and sampling selection bias: firstly, the 347 of 1000 Italian primary care paediatricians who responded could be self-selected and did not reached their potential (34,7%). Secondly, female paediatricians contributed disproportionately to the respondent data set (68% vs. 32%).

Furthermore the “population of preterm infants <34 weeks of gestational age” could be considered a heterogeneous group.

## Figures and Tables

**Table 1:** Timing of introduction of the single food groups in months.

	Corrected Age Mean (SD) [Median], Months	Chronological Age Mean (SD) [Median], Months
Fresh fruit	5.5 (0.58) [5.5]	5.6 (0.50) [6]
Vegetables and Legumes	5.5 (0.5) [5.5]	5.6 (0.5) [6]
Cereals	5.5 (0.5) [5.5]	5.6 (0.5) [6]
Oil	5.5 (1.0) [6]	5.7 (0.8) [6]
Meat	5.6 (0.8) [6]	5.9 (0.6) [6]
Gluten	5.8 (1.2) [6]	6.1 (0.7) [6]
Milk product	7.0 (1.1) [6]	6.4 (1.8) [6]
Fish	7.4 (1.4) [7]	7.6 (1.3) [7.5]
Eggs	8.7 (1.7) [8]	8.5 (1.8) [8.5]
Salt	14.4 (5.2) [12]	16.2 (7.8) [13]
Sugar	16.3 (7.4) [12]	17.1 (6.9) [12]

**Table 2:** Timing of Iron and Vitamin D supplementations.

	Corrected Age Mean [Median], Months		Chronological Age Mean [Median], Months	
	Start	End	Start	End
Iron	1.5 [0]	9.3 [6]	1.5 [1]	9.9 [6]
Vitamin D	0.5 [0]	21.1 [24]	0.6 [0]	20.6 [24]

**Table 3:** Doses of Iron and Vitamin D supplementations.

	Vitamin D Supplementation % of Responders
<400 I.U. day	5%
400 I.U. day	70%
>400; <800 I.U. day	13%
800 I.U. day	10%
No supplementation	2%
	Iron supplementation % of responders
<2 mg/kg/day	30%
2–5 mg/kg/day	58%
>5 mg/kg/day	3%
No supplementation	9%
